# Immunization with Cholera Toxin B Subunit Induces High-Level Protection in the Suckling Mouse Model of Cholera

**DOI:** 10.1371/journal.pone.0057269

**Published:** 2013-02-28

**Authors:** Gregory A. Price, Kim McFann, Randall K. Holmes

**Affiliations:** 1 Department of Microbiology, University of Colorado School of Medicine, Aurora, Colorado, United States of America; 2 Colorado Biostatistics Consortium, Colorado School of Public Health, Aurora, Colorado, United States of America; Instituto Butantan, Brazil

## Abstract

Cholera toxin (CT) is the primary virulence factor responsible for severe cholera. *Vibrio cholerae* strains unable to produce CT show severe attenuation of virulence in animals and humans. The pentameric B subunit of CT (CTB) contains the immunodominant epitopes recognized by antibodies that neutralize CT. Although CTB is a potent immunogen and a promising protective vaccine antigen in animal models, immunization of humans with detoxified CT failed to protect against cholera. We recently demonstrated however that pups reared from mice immunized intraperitoneally (IP) with 3 doses of recombinant CTB were well protected against a highly lethal challenge dose of *V. cholerae* N16961. The present study investigated how the route and number of immunizations with CTB could influence protective efficacy in the suckling mouse model of cholera. To this end female mice were immunized with CTB intranasally (IN), IP, and subcutaneously (SC). Serum and fecal extracts were analyzed for anti-CTB antibodies by quantitative ELISA, and pups born to immunized mothers were challenged orogastrically with a lethal dose of *V. cholerae*. Pups from all immunized groups were highly protected from death by 48 hours (64–100% survival). Cox regression showed that percent body weight loss at 24 hours predicted death by 48 hours, but we were unable to validate a specific amount of weight loss as a surrogate marker for protection. Although CTB was highly protective in all regimens, three parenteral immunizations showed trends toward higher survival and less weight loss at 24 hours post infection. These results demonstrate that immunization with CTB by any of several routes and dosing regimens can provide protection against live *V. cholerae* challenge in the suckling mouse model of cholera. Our data extend the results of previous studies and provide additional support for the inclusion of CTB in the development of a subunit vaccine against *V. cholerae*.

## Introduction

Although there are over 200 serogroups of *Vibrio cholerae* only two, 01 and 0139, are known to cause epidemic/pandemic cholera, which is characterized by acute watery diarrhea [1]. The O1 serogroup contains two biotypes, the classical and El Tor, and two primary serotypes Inaba and Ogawa [2]. The first 6 cholera pandemics were thought to be caused by the classical biotype, and the current 7^th^ pandemic has been primarily caused by the El Tor biotype with the appearance of the 0139 serogroup in 1992 contributing to the current pandemic [2]. Cholera toxin (CT) is the principal virulence factor responsible for the effusive diarrhea associated with severe cholera infection.

CT is an AB_5_ toxin composed of one A polypeptide (CTA) and five identical B polypeptides (CTB). The CTB monomers associate in a non-covalent fashion to form a pentameric ring-like structure [3]. The toxic A subunit is tethered non-covalently to the B subunit via the non-toxic A2 domain which passes through the central pore of CTB [3]. The CTB pentamer serves as the binding domain for CT and binds multivalently to cellular surface receptor G_M1_ ganglioside [4], [5]. CT enters intestinal epithelial cells by endocytosis and is transported to the endoplasmic reticulum by retrograde transport [4]. Cellular intoxication ensues when the A subunit is retrotranslocated into the cytosol and ADP-ribosylates the α-subunit of the heterotrimeric G protein (Gsα), causing a sustained activation of adenylate cyclase and an increase in intracellular adenosine-3′, 5′-monophosphate (cAMP) levels [6]. The resulting rise in intracellular cAMP causes an opening of chloride channels and a net efflux of chloride ions and fluid into the intestinal lumen [7]. The subsequent voluminous watery diarrhea can result in death within a matter of hours of the first symptoms without proper rehydration therapy [1].

Early analysis of CT derived from both the classical and El Tor biotypes demonstrated 3 different variants due to minor sequence differences in the CTB coding region [8]. The classical CTB biotype, genotype 1, was 100% conserved among the classical strains tested [8]. Analysis of El Tor strains demonstrated two different genotypes, 2 and 3 [8]. Recently, 3 new CT genotypes have been discovered along with hybrid El Tor strains expressing the classical genotype 1 CTB [9]–[13]. Dubey et al. tested purified classical and El Tor CTs both *in vitro* and *in vivo* and demonstrated indistinguishable G_M1_ ganglioside binding ability; and, despite minor epitope differences, antisera raised to either one had strong cross-neutralizing activity [14].

Numerous animal studies have demonstrated the toxin neutralizing ability of antibodies to CT and its subunits in protection from CT or live *V. cholerae* challenge [15]–[22]. Human studies using chemically-detoxified CT on the other hand did not show any demonstrable protective efficacy [23]–[25]. One potential limitation of these studies was the use of chemical detoxification to prepare the CT antigen. It has been previously demonstrated that glutaraldehyde detoxification of CT had deleterious effects on toxoid antigenicity [26]. Further, in field trials with CT derived toxoid, only one dose was analyzed; a dose that was insufficient in inducing maximal anti-toxin titers in humans [27]. The link between anti-toxin antibodies and protection from cholera has not been clearly demonstrated in humans, however in breastfed infants there is a correlation between antitoxin antibody titers and development of disease [28], [29]. In addition, a combined killed *Vibrio cholerae* whole-cell and CTB vaccine was demonstrated to have better short-term protective efficacy than whole-cell vaccine alone [30]. Animal models have also demonstrated synergistic protection following vaccination with CTB and somatic antigens [18], [31]–[33].

For the development of a potential subunit vaccine against cholera, two types of immunity are desirable; anti-CT and anti-bacterial. For this study we focused on the toxin neutralization arm of immunity by using recombinant CTB (rCTB) as a vaccine antigen. We previously demonstrated that IP immunization with rCTB elicited a high level of protection against challenge by the virulent *V. cholerae* El Tor strain N16961 in pups reared from CTB or CTB+TcpF immunized mice [34]. For this study, we investigated how route and number of immunizations using a fixed dose of rCTB would influence the protective efficacy in down-stream infant mouse challenges from immunized dams. To this end we immunized adult female mice via intranasal (IN), subcutaneous (SC), and intraperitoneal routes. For IP immunizations 3 groups were immunized with one, two, or three doses of rCTB, respectively. Following immunizations serum and fecal extracts were collected and analyzed for CTB-specific IgG or IgA using quantitative ELISA. The immunized female mice were then mated, and protective efficacy of their CTB-antibody responses were evaluated indirectly by orogastric challenge of their reared pups with *V. cholerae* El. Tor N16961. We demonstrate here that immunization regimens involving varying routes and numbers of doses of CTB can be highly protective in the infant mouse model of cholera. Three parenteral doses of CTB, which elicited the highest serum anti-CTB IgG levels in dams and gave the highest survival rates in their pups, also protected most effectively against weight losses by the challenged pups at 24 hours post infection.

## Results

### Serum and Fecal CTB-specific Antibody Responses

**Figure 1:**
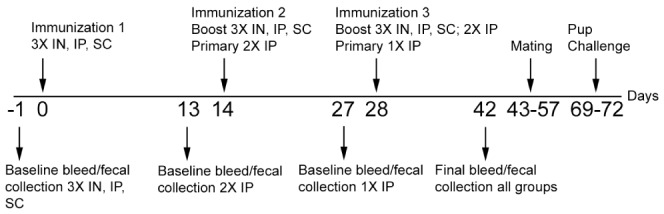
Timeline of immunization and challenge experiments. Immunizations and baseline bleeds/fecal collections were staggered depending on the number of immunizations per group to allow for the final/only immunization(s) to occur on the same day for all groups. All boosts occurred at 14-day intervals, and a final bleed/fecal collection occurred 14 days following the final/initial immunization.

**Figure 2:**
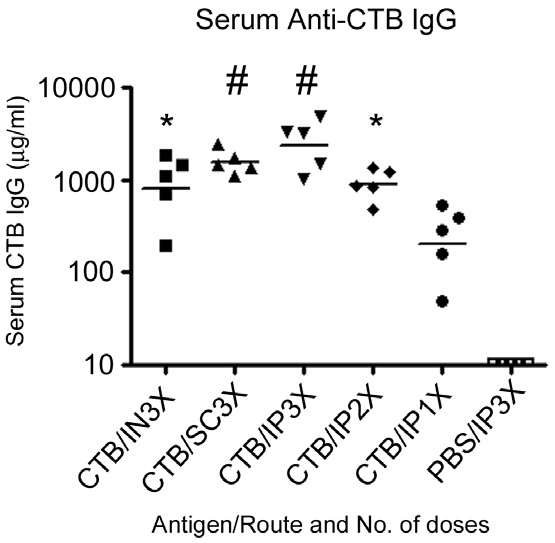
Serum CTB-specific IgG concentrations at 14 days following the final/only immunization via the intranasal (IN), subcutaneous (SC), or intraperitoneal (IP) route. Each symbol represents the serum anti-CTB antibody concentration for an individual mouse. The horizontal lines represent the geometric mean concentration per group (*n* = 5). Statistical differences between groups were analyzed using ANOVA with Tukey-Kramer post-test analysis (# P<0.001 and *P<0.05 versus the CTB/IP1X group).

Groups of 5 female CD-1 mice were immunized by the IN, SC, or IP route with 30 µg of CTB on three occasions at 14-day intervals. Serum and fecal samples were collected one day prior to initial immunization and 14 days following the final/only immunization (See Fig. 1 for timetable). Furthermore, two additional groups of mice received IP primary immunizations on booster days to analyze antibody amounts following one or two doses of CTB (also shown in Fig. 1). Using quantitative ELISA, we analyzed serum and fecal samples for CTB-specific responses in all of these immunization groups. IP and SC immunizations with 3 doses of CTB gave the highest serum IgG anti-CTB antibody amounts with the geometric mean titers in these groups being 2.4 mg/ml and 1.6 mg/ml, respectively (Fig. 2). The IN3X and IP2X immunized groups had similar IgG anti-CTB geometric mean antibody titers of 0.84 mg/ml and 0.90 mg/ml, respectively. IP immunization with one dose of CTB gave an anti-CTB IgG geometric mean titer of 0.22 mg/ml. Immunization with three doses of CTB by the IP, SC, or IN route or two doses of CTB by the IP route generated serum anti-CTB IgG titers that were significantly higher than the group receiving only one dose of CTB IP (*P*<0.001 SC and IP3X; *P*<0.05 IN and IP2X; Fig. 2). All CTB immunized groups had significantly higher CTB-specific IgG antibodies compared to the PBS control group which had no measureable CTB-specific IgG (*P*<0.001; data not shown).

**Figure 3:**
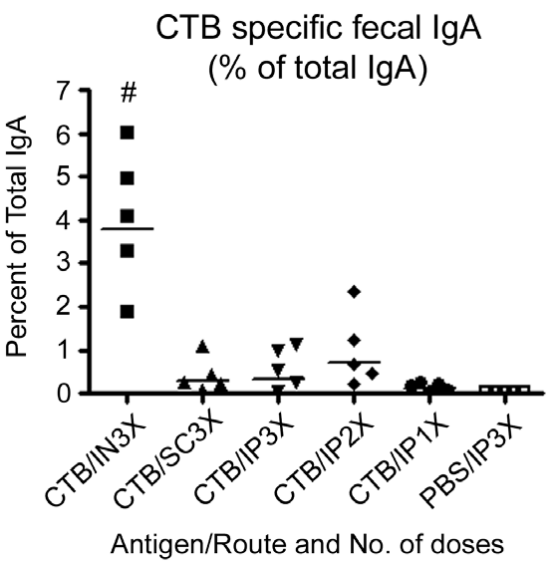
Relative abundance of fecal CTB-specific IgA at 14 days following the final/only immunization via the intranasal (IN), subcutaneous (SC), or intraperitoneal (IP) route. For each fecal extract, the relative abundance of CTB-specific IgA is expressed as a percentage of the total IgA in that fecal extract. Each symbol represents an individual mouse, and horizontal bars represent the geometric mean for that group (*n* = 5). Statistical differences between groups were analyzed using ANOVA with Tukey-Kramer post-test analysis (# P<0.001 versus all other groups).

Fecal extracts were analyzed for CTB-specific IgA levels 14 days after the final/only immunization. In an effort to eliminate sample-to-sample and/or mouse-to-mouse variation, we normalized fecal CTB-specific IgA values by presenting them as percentages of total IgA in the corresponding fecal extracts (Fig. 3). Intranasal immunization was the most potent route for induction of fecal CTB-specific IgA, with values ranging from just under 2% to over 6% of total IgA in samples from individual mice. The geometric mean for the IN group was 3.8% of total IgA, whereas the rest of the immunized groups had CTB-specific geometric means that were detectable but less than 1% of total IgA. The levels measured in the IN immunized group were significantly higher then in all other groups (Fig. 3; *P*<0.001). Total IgA levels in the IN group were not significantly different from total IgA levels in the other groups (data not shown).

### Suckling Mouse Challenge

To investigate whether route of immunization and amount of CTB-specific serum IgG or fecal IgA could predict protection from cholera, we challenged pups born to immunized female mice from each of the immunization protocols described above. Following immunization, all female mice were mated one-to-one with 10-week old CD-1 male mice for 15 days (see Fig. 1 for timeline). Following the mating period, male mice were removed and the females were monitored for birth. Six- to seven-day old infant mice from each cohort of immunized adult female mice were removed from their mothers and either inoculated with a ∼10 LD_50_ dose of *Vibrio cholerae* El Tor strain N16961 or sham inoculated with the AKI media used to grow the *V. cholerae* inoculum. The pups were placed in Petri dishes containing sawdust and placed on 30°C heating pads. The pups were monitored over a 48-hour period for survival. As shown in Table 1, pups born to mothers immunized by all routes and each number of immunizing doses of CTB tested exhibited significant protection from death following challenge with *V. cholerae*. In striking contrast, pups born to PBS-immunized mothers had 100% lethality. Interestingly pups from mothers immunized IN3X, which had the highest fecal anti-CTB IgA titers and robust serum anti-CTB IgG titers, exhibited the lowest survival rate among the immunized groups. This unexpected result was most likely due to an excess mortality of 100% among the pups reared from one of the IN immunized dams. Although the pups from that one dam were healthy prior to challenge with *V. cholerae*, as assessed by visual inspection and body weight, 80% of that litter of pups died prior to the 24-hour time point, and all died by 48 hours. The 80% death rate at 24 hours for that litter was higher than for pups from the PBS-immunized controls, in which the highest mortality at 24 hours from pups reared from any specific dam was 50%. Anti-CTB antibody concentrations did not provide a likely explanation for the results observed with the anomalous litter, since the corresponding dam had the highest serum anti-CTB IgG concentration as well as the second highest fecal anti-CTB IgA level among all of the immunized mice. Because of the 100% mortality among pups reared from the above-mentioned dam, the overall protective efficacy for the IN3X group was significantly less than for SC3X and IP3X groups (*P*<0.05.; Table 1). However, if the one anomalous group of pups is removed from analysis, the protective efficacy from the remaining IN immunized cohort was 27/32 survivors, with a robust 84% protective efficacy that was not significantly different from any of the other immunized groups (*P*>0.05). Pups from mice immunized SC3X with CTB were 100% protected from death, though there were 10–12 pups fewer in this challenge group compared to the IN3X, IP3X, and IP2X groups. The protective efficacy for the SC3X group was not significantly greater than for the IP3X group (*P*>0.05), but it was significantly greater than for the IP2X or the IN3X group (*P*<0.05;Table 1). The protective efficacy for the IP3X group was significantly greater than for the IN3X group (*P*<0.05;Table 1), but the differences in protective efficacy between the IP3X, IP2X and IP1X groups were not statistically significant (*P*>0.05;Table 1). The protection in all CTB-immunized groups was significantly greater than for the PBS-immunized control group (*P*<0.0001).

**Table 1:** Infant mouse challenge with 10 LD_50_ *V. cholerae* N16961.

Challenge Group (route/# vaccinations)	Survivors/total (% survival)
Sham infected control*	31/31 (100%)
PBS control (IP3X)	0/42 (0%)#
CTB (IN3X)^1^	27/42 (64%)@
CTB (SC3X)	31/31 (100%)
CTB (IP3X)	37/41 (90%)
CTB (IP2X)	34/43 (79%)%
CTB (IP1X)	27/31 (87%)

* Infant pups were from a mixture of immunization groups and challenged with AKI media only.

# Significantly different from all CTB immunized groups (*P*<0.0001).

@Significantly different from all CTB immunized groups except IP1X and IP2X (P<0.05).

% Significantly different from CTB/SC3X (*P*<0.05).

1 Pups from one dam were highly sensitive to *V. cholerae* infection and all died by 48 hours. The protective efficacy of the remaining IN cohort was 27/32 (84% survival; not significantly different from the other CTB immunized groups).

### Evaluation of Weight Loss as a Potential Surrogate Marker of Protective Efficacy in the Infant Mouse Model

Because infant mice are susceptible to cholera infection, they develop severe diarrhea following challenge with *V. cholerae* if they lack protective immunity. This loss of fluid from diarrhea can be measured over the course of infection as % weight loss compared to T = 0 levels. By comparing experimental groups with sham-inoculated groups at various time intervals, normal weight loss associated with separating the pups from their dams can be differentiated from pathological weight loss due to the diarrheal disease caused by infection with *V. cholerae*. We used this approach to investigate whether weight loss at 24 hours predicted death by 48 hours and if weight loss could potentially be used as a surrogate marker for protective immunity in the infant mouse model of cholera. To this end, all pups used as experimental animals or controls in the infant mouse cholera model were weighed to an accuracy of one hundredth of a gram at times 0, 24, and 48 hours, and the percentage weight loss was determined for the 0–24 hour, 0–48 hour, and 24–48 hour periods. The pup weights did not differ significantly for any of the groups at T = 0 (data not shown). Each pup was numbered with a marking pen to uniquely identify each individual. For animals that died prior to a time point, the carcass weight was measured at the time of discovery and included with the group data for the next time point. For the final 48-hour time point, the data included both survivors and the pups that died during the final 24 hours.

**Figure 4:**
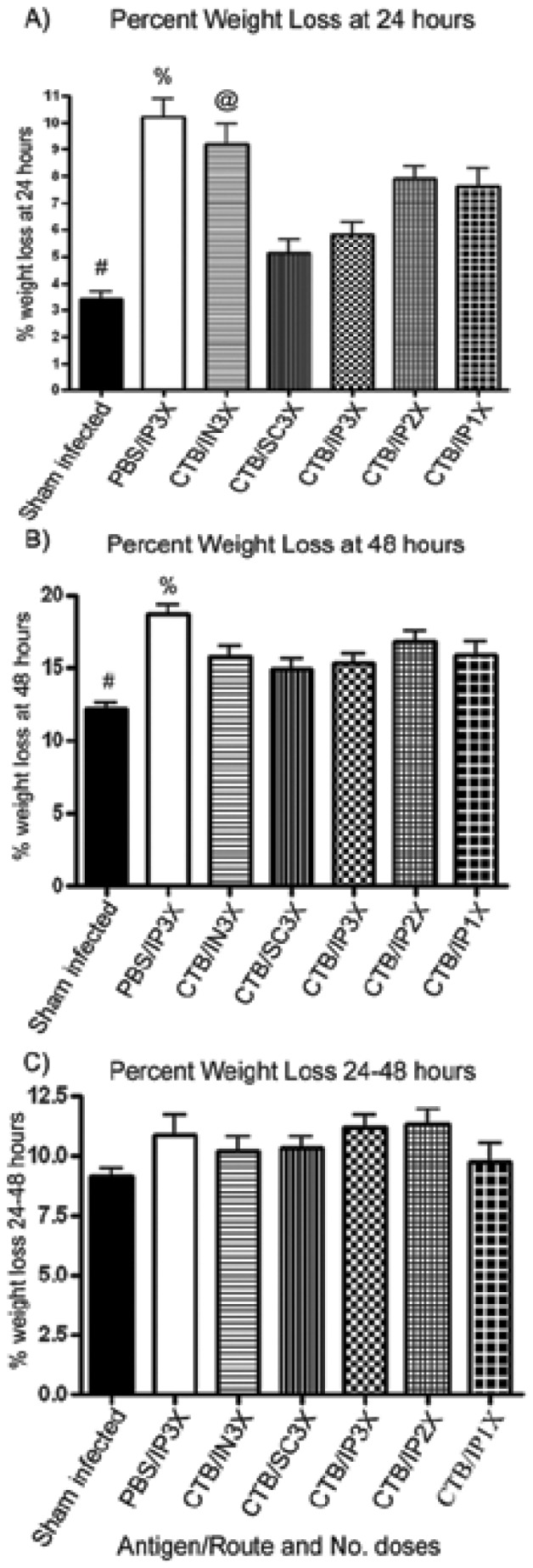
Analysis of average weight loss at 24 hours, at 48 hours, and during the 24- to 48-hour interval post infection with *V. cholerae*. Individual mouse pups were weighed at 24 and 48 hours post-infection and their weight loss was compared to their initial weight at T = 0 (A and B) or 24 hours (C). Error bars represent the SEM. Statistical differences between groups were analyzed by mixed models repeated measures analysis with Tukey-Kramer post-hoc test analysis. Symbols 4 A+B, ^#^ and ^%^ see Table 2 for statistical comparisons, and ^@^ P<0.0001 vs IP3X and P = 0.0022 vs SC3X groups.

As can be seen in figure 4A, there was a striking difference in weight loss at 24 hours between the sham-infected group and the PBS immunized controls, demonstrating that the *V. cholerae* inoculum was virulent and caused rapid weight loss in the unprotected PBS-immunized group (*P*<0.0001; Table 2). Though protection from death by 48 hours was high in pups from all CTB immunized groups (Table 1), only pups from the IP3X and SC3X groups exhibited weight losses at 24 hours that did not differ significantly from the sham-infected group (*P*>0.05; Table 2). Furthermore, the IP3X and SC3X dams had the highest mean anti-CTB serum IgG concentrations, and their pups exhibited the highest survival rates at 48 hours (Fig. 2 and Table 1). Conversely, pups from the IN3X immunization group had the lowest survival at 48 hours (Table 1) and exhibited the greatest weight losses at 24 hours among all of the CTB immunization groups (Fig. 4A). At 24 hours the IN3X group showed significantly greater weight loss than both the IP3X and SC3X groups (P<0.0001 and 0.0022 respectively). These findings were influenced by excessive weight losses and 100% mortality among the pups reared from one mouse as noted above. Removal of these pups from the data analysis demonstrated that the rest of the pups from the IN immunized cohort had weight losses similar to the IP2X and IP1X immunized groups (data not shown).

**Table 2:** Percent weight loss statistical comparisons at 24 and 48 hours (Sham infected/PBS immunized controls vs. other challenge groups).

Sham infected/PBS Controls	Challenge Group Comparison	Time (hrs)	AdjustedP-value	Time (hrs)	AdjustedP-value
Sham Infected	PBS/IP3X	24	<0.0001	48	<0.0001
Sham Infected	CTB/IN3X	24	<0.0001	48	<0.0001
Sham Infected	CTB/SC3X	24	0.9082	48	0.3028
Sham Infected	CTB/IP3X	24	0.4134	48	0.0023
Sham Infected	CTB/IP2X	24	0.0003	48	<0.0001
Sham Infected	CTB/IP1X	24	0.0042	48	0.0007
PBS/IP3X	CTB/IN3X	24	0.9970	48	0.9226
PBS/IP3X	CTB/SC3X	24	<0.0001	48	0.0012
PBS/IP3X	CTB/IP3X	24	<0.0001	48	0.0158
PBS/IP3X	CTB/IP2X	24	0.2931	48	1.0000
PBS/IP3X	CTB/IP1X	24	0.2496	48	0.1882

By 48 hours, all but the SC3X group had significantly greater weight losses than the sham-infected control (Table 2). However both the IP3X and SC3X had significantly lower weight losses compared to the PBS immunized control group (P = 0.0158 and 0.0012 respectively; Table 2), which had no survivors. Calculation of weight losses for each group between 24 and 48 hours showed there were no significant differences between any of the groups (Fig. 4C), and the substantial amount of weight being lost in the final 24 hours both in control and infected animals was presumed to be due primarily to the starvation conditions. Cox Regression showed that weight loss at 24 hours strongly predicted death by 48 hours, HR 318.35 (92.34–1097.53). There were 74 events and 187 pups censored. This analysis suggests that protection against weight loss in the first 24 hours is an important factor in the ability of infected mice to survive to the 48-hour endpoint. Furthermore, the mean weight loss at 24 hours among the 187 pups in Table 1 that survived to the 48-hour endpoint was 5.63 percent (95% Confidence Interval of 5.21 percent to 6.05 percent), and this value differed significantly (*P*<0.0001) from the mean weight loss at 24 hours of 11.37 percent (95% Confidence Interval of 10.41 percent to 12.33 percent) among the 74 pups that died by 48 hours.

## Discussion

We demonstrated here that recombinant CTB is a potent immunogen and generated strong serum CTB-specific IgG responses in adult mice following SC, IP, or IN immunization. Furthermore, CTB immunization of dams provided significant protection for their pups against death following orogastric challenge with a 10 LD_50_ dose of *Vibrio cholerae* strain N16961. These results are not without precedent, as numerous studies with various animal models have demonstrated protection against diarrhea using hyperimmune sera or direct vaccination with cholera toxin, detoxified cholera toxin, or cholera toxin subunits [15]–[22]. Nevertheless, the correlations of immunization routes, the number of immunizing doses, and the amounts of CTB-specific IgG or IgA with protection have not previously been studied in detail in the suckling mouse model of cholera.

Using a fixed dose of CTB, we were able to demonstrate a dose-response relationship between serum anti-CTB IgG amounts and administration of one, two, or three immunizing doses of CTB via the IP route. Immunization with three doses of CTB administered via the SC route generated serum CTB-specific IgG in amounts comparable to three doses via the IP route. We also added an IN immunization group with the goal of generating high fecal anti-CTB IgA levels. Fecal anti-CTB IgA antibodies were present in all CTB-immunized mice regardless of the immunization route, but the highest levels were found in mice immunized by the IN route. Only total CTB-specific IgA was measured in each fecal extract, and the fractional amount of sIgA was not determined All CTB-specific IgG and IgA antibodies present in the pups are presumed to have been produced by the immunized dams, but the routes by which those antibodies were delivered to the pups were not directly demonstrated in this study.

While the IN3X regimen was immunogenic, animals in this group exhibited greater variability in CTB-specific serum IgG and fecal IgA antibody responses than animals in the SC3X or IP3X immunization groups. This outcome is not surprising, because exhalation, inhalation, spillage, or swallowing could cause greater mouse-to-mouse or dose-to-dose fluctuations in antigen delivery by the IN route compared to the SC or IP route. Although the fixed-dose of CTB used in this study would be considered high in relation to previous human studies [23]–[25], it was well tolerated in all mice tested, and no apparent adverse-effects were seen in any of the mice following vaccination by any of the routes tested.

We utilized infant mice from CTB-immunized experimental mothers, and PBS-immunized control mothers, to evaluate protective efficacy following challenge with a 10 LD_50_ lethal dose of N16961 *Vibrio cholerae*. Infant mice develop severe diarrhea following orogastric challenge with *V. cholerae* [35]. In addition, cholera-specific antibody can be transferred from immunized dams to their pups in utero and by suckling [36]–[38]. Therefore, challenging mouse pups from immunized dams with live *V. cholerae* can be used as an indirect method to demonstrate protective efficacy of a vaccine regimen. In this study, immunization with CTB by all regimens tested provided a high and statistically significant degree of protection of pups from death within 48 hours post challenge. However, we were able to show that immunization with three doses of CTB by the SC or IP route elicited the highest mean anti-CTB serum IgG antibody concentrations in dams, which translated into the highest survival rates (100% and 90%, respectively) and the lowest percentage weight losses in the challenged pups at 24 hours (not significantly different from the percentage weight loss in the sham-infected controls). Although the IN3X, IP2X, and IP1X groups were well protected against death, weight loss analysis demonstrated that these groups were not as strongly protected against weight loss as the SC3X and IP3X groups. Taken together with the data in Figs. 2 and 3, these findings suggest that the amount of anti-CTB antibody needed to prevent death of pups by 48 hours is less than the amount needed to confer maximum protection against *V. cholerae-*induced weight loss in the infant mouse model of cholera. This study highlights the added value of measuring weight loss in additional to survival. Without the weight loss analysis used here, it would not have been apparent that protection against *V. cholerae*-induced diarrhea was substantially greater for the IP3X and SC3X groups than for the IP1X and IP2X groups.

Surprisingly the IN3X immunization group, which had high serum CTB-specific IgG concentrations and the highest fecal anti-CTB IgA antibody levels in dams, had the lowest protective efficacy in challenged pups among all of the immunized groups. As mentioned previously in the results section, this outcome was strongly influenced by the anomalous responses of pups from one IN-immunized mother. Those specific pups exhibited rapid weight loss at 24 hours and 100% mortality by 48 hours, in spite of that dam having the highest serum CTB-specific IgG and the second highest fecal CTB-specific IgA levels in the IN immunized cohort. Removal of these pups from data analysis demonstrated that the rest of the pups from the IN-immunized cohort were well protected from death (84%, which was not statistically different from any of the other CTB immunized groups). It is unclear why the pups from the one specific IN immunized dam developed more severe disease prior to 24 hours than pups from all the PBS immunized controls. The mouse strain used in this study was the outbred mouse strain CD-1 which has a large degree of mouse-to-mouse genetic variation [39]. It is possible that an unknown mouse genetic factor may have contributed to the high mortality rate in the pups from this one dam in the IN3X immunization group. In humans severe cholera is prevalent in blood group O humans or those with a variant in the promoter region of the *LPLUNC1* gene [40], [41]. Because the rest of the pups from the IN immunized cohort were well protected from the *V. cholerae* challenge, we consider this to be a plausible hypothesis.

Weight loss is a sensitive tool to help measure vaccine efficacy and the overall health of immunization cohorts over the course of infection in the infant mouse model of cholera, as we demonstrated previously [34]. Results of the current study confirmed and extended those observations, and Cox regression analysis demonstrated that weight loss at 24 hours predicts death within 48 hours. Nevertheless, the confidence interval for HR was wide, reflecting considerable variation in weight loss at 24 hours post challenge among pups from dams within individual immunization cohorts. For that reason, we are not yet able to identify a specific amount of weight loss at 24 hours that can serve as a reliable surrogate marker for vaccine efficacy in the infant mouse model of cholera. Future testing of additional variables (such as more frequent measurements of weight of pups, more accurate determinations of time of death, adjustments in size of the challenge dose of *V. cholerae*, etc.) is therefore warranted in a continuing effort to establish whether weight loss can be validated as a surrogate marker to replace death as an endpoint for use in the infant mouse model of cholera. In any case, this study shows that weight loss analysis complements survival analysis and provides very useful information about the physiological responses and overall health of pups from immunized dams following orogastric challenge with *V. cholerae*.

In humans, correlates of protection from cholera are still poorly defined, but natural infection can lead to long-term protection [42]. Secretory cholera-specific gut IgA is considered important in protection from cholera as IgA is the dominant antibody isotype in the human gut [43]. IgG, however, is also found in the gut, and gut IgG levels have been demonstrated to increase following cholera infection [44], [45], including duodenal IgG levels to CTB and LPS [46]. Mounting evidence suggests that development of memory B cells following natural infection may play a key role in long-term protection against *V.* cholerae, as serum and gut anti-cholera antibodies quickly wane following infection [46]–[48]. Memory B and antigen secreting cells in the serum and duodenum persist following natural infection after antigen-specific antibody amounts return to baseline levels [46], [47], [49], [50]. Recently it was demonstrated that cholera LPS-specific IgG memory B cells in the sera were associated with a reduced risk of cholera infection [48], suggesting a role for both IgA and IgG in protecting humans from cholera. In the suckling mouse model of cholera, protection is elicited by antigen-specific antibodies obtained from the immunized dam [38]. IgG is likely to play a major role in protection of infant mice due to the fact that the major antigen-specific antibody isotype in both sera and stomach contents is IgG [37], [51]. IN immunization of dams can lead to antigen-specific IgA being present in both the stomach contents and sera of reared pups, but at levels much lower than antigen-specific IgG [37]. Furthermore, it was previously demonstrated that IP immunization of dams with *V. cholerae* outer membrane vesicles could prevent *V. cholerae* colonization of reared infant mice in the absence of detectable antigen-specific IgA in the sera or stomach contents [37], [51]. In this study the induction of CTB-specific IgA in the IN immunized group did not appear to enhance protection in the downstream infant mouse challenges, suggesting that CTB-specific IgG is sufficient for optimal CT neutralization. This is not without precedent as human cholera convalescent immunoglobulins purified from sera and milk demonstrated serum derived IgG to be superior at neutralizing CT compared to milk purified IgA [52].

Because viable counts of *V. cholerae* in the intestine were not analyzed in this study, it was not possible to determine whether the protection afforded by CTB immunization was due to blocking the pathophysiological effects of CT produced during infection, eliminating *V. cholera*e from the small intestine due to mechanical and/or immune mechanisms, or both. However, for potential development of a multivalent subunit vaccine against cholera, we clearly demonstrate that production of anti-CT antibodies can be an important component of protective immunity, as CT is the primary direct cause of the massive watery diarrhea associated with severe cholera. However, with the emergence of new CTB genotypes and hybrid strains expressing the classical CTB genotype [9]–[13], a broadly protective subunit vaccine may require the inclusion of more than one CTB genotype. We recently demonstrated that mice immunized with either the El Tor or classical CTB demonstrated significantly lower anti-CTB antibody amounts when the heterologous CTB was used in quantitative ELISAs [34]. Furthermore in infant mouse challenges, pups born to dams immunized with a TcpF holotoxin-like chimera containing the classical CTB variant, had poorer protective efficacy when challenged with the El Tor strain N16961 than pups born to dams immunized with equimolar amounts of El Tor CTB and TcpF or the El Tor CTB alone [34]. This suggests that the minor sequence variations between the classical and El Tor CTB genotypes affect immunodominant epitopes important for antibody-mediated CT neutralization.

Several observations support the conclusion that immune responses against CT, by themselves, are unlikely to provide highly effective protection against cholera; human studies have failed to demonstrate any strong protection against cholera following immunization with toxoid [23]–[25]. Blocking the effects of CT may slow the progression of disease without preventing it, as demonstrated in a human challenge study [24]. In addition, without a significant decrease in fecal shedding of *V. cholerae*, person-to-person spread is possible, which would be an undesirable situation in epidemic cholera. Therefore, targeting colonization factors to block colonization of the small intestine by *V. cholerae* is also likely to be an important component of protective immunity. It has been demonstrated in animal models that CTB and somatic antigens act in a synergistic fashion to promote vaccine efficacy [18], [31]–[33]. In human studies the inclusion of CTB in whole cell (WC) vaccines gave better short-term protection than in groups immunized WC alone [30]. The goal for further development of an effective subunit vaccine against cholera would therefore be two-fold: block the action of cholera toxin and prevent intestinal colonization by the bacteria. This study not only demonstrates the highly protective efficacy of CTB in the suckling mouse model of cholera but also serves as a baseline for future experiments using CTB in combination with other *V. cholerae* antigens in the development of an efficacious subunit vaccine.

## Materials and Methods

### Ethics Statement

All procedures involving immunization and breeding of adult CD1 mice and challenge of pups with *Vibrio cholerae* N16961 in the infant mouse model of cholera were approved by the University of Colorado Denver Animal Care and Use Committee. The studies were done under protocol 33701206(10)F which was initially approved on 10/11/2006 and protocol 33709(11)1E which was initially approved on 11/4/2009. This Institution has an Animal Welfare Assurance on file with the Office of Laboratory Animal Welfare. The Assurance number is PHS A3269-01 (09/31/2011). This Institution is accredited by the Association for Assessment and Accreditation of Laboratory Animal Care (AAALAC) - File Number 00235.

### Construction of Recombinant CTB Expression Plasmids

Genomic *Vibrio cholerae* N16961 DNA was used to PCR amplify the genes encoding the mature cholera toxin B subunit. The forward primer, CGC**TGGCCA**CACCTCAAAATATTACTG, contains an MscI site (shown in bold) and amplifies from the 5′ end of the coding region for mature CTB. The reverse primer, TTT**CTCGAG**TTAATTTGCCATACTAATTGC, contains an XhoI site (shown in bold) and amplifies from the 3′ end of the coding region for CTB. This amplicon was digested with MscI and XhoI and ligated into the MscI and XhoI cut pET-22b(+) (EMD Biosciences, Gibbstown, NJ ) in frame with the *pelB* leader sequence, creating the expression plasmid pGAP20A. The ampicillin resistant pGAP20A expression plasmid was restriction digested with XbaI and XhoI and the piece containing the ribosomal binding site and the *pelB-ctb* gene was agarose gel purified. This fragment was then ligated into pET-28b(+) (EMD Biosciences, Gibbstown, NJ ) creating the kanamycin resistant rCTB expression plasmid pGAP20K. The host strain used for rCTB expression was BL21(DE3).

### Recombinant CTB Expression/Purification

Recombinant CTB was expressed in half-liter cultures containing TCYM pH 7.5 (Tryptone 1%, NaCl 0.5%, yeast extract 0.5%, casamino acids 0.1%, MgSO4 0.2%) and 100 µg/ml kanamycin. Cultures were grown at 37°C with shaking at 250 rpm until the optical density at 600 nm reached ∼3.0. Cultures were then shifted to 22°C with shaking at 250 rpm, grown for 30 minutes to 1 hour, induced by addition of IPTG at a final concentration of 0.1 mM, and then grown overnight (∼16–18 hours) at 22°C with shaking at 250 rpm.

Following overnight induction the cells were pelleted and resuspended in 50 mM NaH_2_PO_4_, 300 mM NaCl, pH 8.0. To obtain soluble extracts, 2% Elugent (EMD Biosciences, Gibbstown, NJ ), 50 ug/mL lysozyme, and protease inhibitors (Sigma, St. Louis, MO) were added and incubated with shaking at 4°C for 1 hour. Solubilized extracts were subjected to sonication on ice until no longer viscous. The insoluble debris was removed by centrifugation at 25132×g for 15 minutes at 4°C. Talon metal affinity resin (Clontech, Mountain View, CA) was added to soluble extracts to permit native rCTB to batch-bind to the resin at room temperature for 30 minutes. Following batch-binding, the Talon resin was washed with 75 bed volumes of the above phosphate buffer. The rCTB was eluted from the column with the above phosphate buffer containing 250 mM imidazole. Following elution, rCTB was then dialyzed against 25 mM potassium phosphate buffer pH 6.6 overnight at 4°C. The dialyzed rCTB was subjected to centrifugation to remove precipitated material and then passed through a 0.45 µM syringe filter. A secondary ion-exchange purification step was conducted using cation-exchange POROS® 20 HS resin (Applied Biosystems, Carlsbad, CA). Bound rCTB was eluted using a linear gradient of 0.5 M NaCl in the above potassium phosphate buffer. The purified rCTB was then dialyzed against 1X PBS pH 7.4 and stored at −80°C until time of use. Analysis of the rCTB by SDS-PAGE revealed no contaminating proteins.

### Immunizations and Sample Collections

Female CD-1 mice, 6–8 weeks old, were purchased from Charles River Labs (Wilmington, MA). Mice were housed in microisolator cages and given food and water ad libitum. Groups of 5 mice were immunized either intraperitoneally, subcutaneously, or intranasally with 30 µg of rCTB per immunization without adjuvant. For the parenteral routes of immunization, the mice were immunized with a volume of 100 µl per dose. For intranasal immunizations, the mice were first anesthetized with isoflurane, then 30 µg of rCTB in a 10 µl volume was delivered to the anesthetized mice using a P-10 pipette (5 µl per naris). Groups of mice that received multiple immunizations were immunized at 14-day intervals. Blood and fecal samples were collected one day prior to initial immunization, and 14 days following the last/only immunization (Figure 1 shows immunization/sample collection timelines). Sera were obtained by submandibular bleeding using 5 mm Goldenrod Animal Lancets (Medipoint, Inc., Mineola, NY), and stored at −20°C. Fecal samples were obtained from individual mice, weighed and re-suspended at 5–10 µl/mg of feces in 1X PBS pH 7.4 containing 50 mM EDTA, 0.1 mg/ml soybean trypsin inhibitor and 1 mM phenylmethanesulfonyl fluoride (PMSF). Fecal pellets were vortexed until fully macerated, and then the insoluble material was pelleted by centrifugation. The clarified fecal extracts were stored at −80°C.

### Quantitative ELISA

Sera and fecal extracts were assayed for amounts of CTB-specific antibodies using quantitative ELISA methods similar to previously published procedures [34], [53]. Each 96-well ELISA plate contained either IgG or IgA standards generated with serial dilutions of a mouse reference serum (Bethyl Laboratories) in some wells, and unknown samples in other wells. The standard curves for IgG or IgA immunoglobulins were generated by coating individual wells with 100 µl of goat anti-mouse IgG- or IgA-specific capture antibodies (Bethyl Laboratories, Montgomery, TX) diluted to 1 µg/ml in carbonate buffer (0.015 M Na_2_CO_3_, 0.035 M NaHCO_3_, pH 9.6), and IgG or IgA from the mouse reference serum was subsequently captured, detected with isotype-specific goat anti-mouse peroxidase-conjugated antibodies, and developed using Sigmafast™ OPD substrate (Sigma, St.Louis, MO), as described in more detail subsequently in the assays for anti-CTB antibodies. To measure the anti-CTB isotype-specific antibodies in the unknowns, CTB was coated in separate wells of the 96-well microtiter plate by diluting rCTB to 1 µg/ml in borate-buffered saline (0.05 M boric acid, 0.0012 M sodium tetraborate decahydrate, 0.1 M NaCl, pH 8.2) and adding 100 µl per well. ELISA plates were then incubated at 4°C overnight to allow for antigen coating. Following coating, plates were washed two times with 1X PBS pH 7.4 containing 0.05% Tween 20 and blocked for 1 hour at 37°C with blocking buffer (1X PBS pH 7.4, 5% horse serum, 0.05% Tween 20). Sera or fecal extracts were diluted appropriately in blocking buffer and serially diluted 2-fold in the 96-well plates and incubated overnight at 4°C. The plates were then washed three times with the above wash buffer, and goat anti-mouse peroxidase-conjugated isotype-specific antibodies (Sigma, St.Louis, MO) were used to detect captured CTB-specific IgG or IgA antibodies. Plates were incubated for 2–4 hours at room temperate and then washed three times with above wash buffer. The plates were developed using Sigmafast™ OPD substrate (Sigma, St.Louis, MO), and the reactions were allowed to proceed for 30 minutes in the dark. The reactions were stopped by the addition of 3 M HCl, and the plates were read at 490 nm using a Bio-Tek® Synergy Ht microplate reader (Winooski, VT). The concentrations of IgG or IgA anti-CTB antibodies in the unknown samples were interpolated, using KC4 v3.4 software (Bio-Tek®, Winooski, VT), from the corresponding standard curves for IgG or IgA. Samples were assayed at least twice, each dilution in duplicate, and at 2 dilutions per plate. All samples had a within plate and plate-to-plate coefficient of variation of ≤15%. To measure total IgA in each fecal sample 96-well ELISA plates were coated with goat anti-mouse IgA capture antibodies (Bethyl Laboratories, Montgomery, TX ) diluted to 1 µg/ml in the above carbonate buffer and 100 µl was added to individual wells. The rest of the assay was conducted as described above and unknowns were interpolated from a standard curve generated on each plate with the mouse reference serum.

### Infant Mouse Model

For the preparation of the *V. cholerae* El Tor N16961 inoculum, frozen stocks were streaked onto nutrient agar plates and incubated at 30°C for 22–24 hours. Single colonies were inoculated into 40 ml samples of freshly prepared AKI broth (1.5% Bacto-peptone, 0.4% yeast extract, 0.5% NaCl, 0.3% NaHCO_3_) in sterile 50 ml conical tubes and placed at 30°C without shaking for 15–16 hours. Bacteria were then pelleted by centrifugation at 7650×g for 10 minutes at room temperature. The bacteria were then re-suspended in fresh AKI medium to an OD_600_ of 0.34 which corresponded to ∼1.1×10^9^ CFU/ml. This stock was then diluted in AKI media containing 0.01% Evan’s Blue Dye to create the 10 times lethal dose 50 (10 LD_50_) inoculum. The LD_50_ of *V. cholerae* N16961 determined in preliminary experiments using inocula of increasing CFUs based on half-log increments. Groups of 10 6-day-old mouse pups were challenged with an inoculum and monitored for survival over a 48-hour time window. The LD_50_ was extrapolated using the method by Reed and Muench [54] and was determined to be ∼7×10^5^ CFU. The 10 LD_50_ inoculum was plated on LB agar plates, which were incubated overnight at 30°C to confirm the expected number of viable bacteria in each sample.

Immunized females were mated 1-to-1 with 10-week old male CD-1 mice for 15 days (see Figure 1 for timeline). The males were removed and the females were monitored for birth. At 6 or 7 days of age the pups were removed from their mothers for 3 hours prior to challenge. Immediately prior to inoculation the pups were weighed to one hundredth of a gram and numbered on the back with a permanent marker for individual identification. For inoculation a 1 ml syringe was fitted with a 23-gauge needle and one inch PE50 tubing placed over the needle. Each pup was inoculated intragastrically by oral gavage with a 10 LD_50_ inoculum in 100 µl, or 100 µl of AKI broth alone for the sham infected group. The addition of Evan’s blue dye in the media allowed for visualization of inoculum deposition into the stomach. Pups were housed in large plastic Petri dishes (100×25 mm) containing sawdust bedding and placed on a 30°C warming pad. The pups were monitored for survival over the course of 48 hours and also weighed at 24 and 48 hours. Carcass weights were taken at the time of discovery from pups that perished over the course of the infection and those weights were included at the next time point. In separate preliminary experiments we found that carcass weights remained relatively static over the course of six hours (data not shown).

### Statistical Analysis

Statistical analyses were performed using GraphPad PRISM® 4 software and SAS 9.3. Statistical differences between groups for anti-CTB antibody amounts was analyzed using ANOVA with the Tukey-Kramer post-hoc test and percent weight losses was analyzed using mixed model repeated measures with the Tukey-Kramer post test since measurements were taken at 24 and 48 hours after baseline. Statistical comparisons between groups for protection from death in the infant mouse model were performed using contingency tables and *P* values were obtained using Fisher’s exact test. A Bonferroni adjusted *P* value less than 0.05 was considered significant. Cox Regression was used to determine if percent weight loss at 24 hours predicted death. Data are presented as HR and 95% CI.
